# Early Tuberculosis of the Kidney

**Published:** 1908-04-25

**Authors:** 


### Medicine.

### EARLY TUBERCULOSIS OF THE KIDNEY.

The diagnosis of a tuberculous lesion in a kidney is by no means easy; evidence is accumulating to show that the disease is much more common than was formerly thought, and in this respect it is comparable to tuberculosis of the bladder, the existence of which has been demonstrated again and again by the use of the cystoscope in cases in which formerly its presence would not have been suspected. The most satisfactory conclusion that can be drawn from these facts is that tuberculous lesions, both of the kidney and of the bladder, get well spontaneously far more than was once supposed.

Tuberculosis of the kidney may or may not be associated with a similar affection of the bladder. In the latter case the symptoms are chiefly vesical and consist mainly of hypogastric pain when the bladder becomes at all distended, with consequent great frequency of urination. It is not of such cases that we would speak here, but rather of patients in whom the trouble is almost or quite entirely renal. The symptoms in the later stages, when the lesion can be diagnosed with comparative ease, are dull pains in one or other loin, loss of weight, the passage of pus in an acid urine almost constantly, together with blood from time to time, and the presence of a fulness, or even an actual mass, in the region of the affected kidney. When the condition has reached this stage it has already been in existence for months or longer; it should be one's object to detect the mischief much earlier than this.

The questions which immediately arise are: What are the symptoms of renal tuberculosis in its earlier stages? What general examinations may assist the diagnosis? What examinations of the urine are best calculated to reveal the diagnosis?

### Symptoms.

The earliest symptom may be hæmaturia. In a great many cases a microscopical examination of the urine will detect the red blood corpuscles long before the colour of the urine itself attracts attention to the blood. Sooner or later, however, there is sufficient hæmaturia for the patient's own notice to be attracted to it, and this at a time when the general health may still be good. It by no means follows, of course, that such hæmaturia indicates a tuberculous lesion of necessity: there may be a small calculus, a condition of oxaluria, a local injury, a nephritis, a growth, or other things to account for it. But an apparently causeless hæmaturia is so often a very early symptom of renal tuberculosis that it is wise to investigate every case of the kind.

The next most common symptom, and one which may sometimes be the first to attract attention, is the deposition in the urine, on standing, of small quantities of pus. Slight pyuria is never a thing to be passed over lightly, particularly in a young adult with a tuberculous family history; if no adequate cause for it can be found in other ways, guinea-pig inoculations should be resorted to as described presently.

The third symptom of renal tuberculosis is failure of nutrition, together with discomfort in the region of one or other kidney. By the time that these are obvious the renal lesion is already past its first stages, and there are probably large areas of caseation in the organ. Intermittent pain, more or less colicky in character, is in some cases due to partial obstruction of the ureter with pus, and in others to cicatrisation of the upper part of the ureter with accumulation of urine or pus above the stricture; either of these may occur before the kidney has become greatly destroyed by the caseating process, but neither can be termed a symptom of the trouble in its earliest stages.

When pain in the kidney has become severe the lesion has reached a late stage, and when there is frequency of urination the probability is that the bladder has become involved. Frequency of micturition in these cases is essentially a vesical and not a renal symptom. One kidney may be much diseased without there being any increased frequency of urination at all.

### Methods of Investigation.

Now, cystoscopy and segregation are fairly formidable procedures, whatever experts may say to the contrary. No doctor would himself submit to either operation if less serious measures suffice to clinch the diagnosis. The same applies to the tuberculo-ophthalmic reaction of Calmette. This is distinctly uncomfortable, though, like cystoscopy and segregation, it is invaluable in certain cases. What steps other than the above can be adopted which are less troublesome to the patient and often quite as serviceable in determining the diagnosis? They are: general microscopical examination of the centrifugalised deposit from the urine; special examination of stained films of the deposit for tubercle bacilli; and injections of the urine into guinea-pigs.

Little need be said about the first two of these, for they are so obvious. If the general microscopical examination reveals fragments of calculi, ova of *Bilharzia hæmatobia*, hyaline and granular renal tube casts, or particles of new growth, the cause for the hæmaturia has been found. If, on the other hand, the general microscopical examination shows red blood corpuscles and pus cells in an acid urine, without any obvious cause for their presence, the next step is to stain films of the deposit by the Ziehl-Neelsen method. There are fallacies in this examination, as has already been pointed out in this journal, but, none the less, in quite half the cases of renal tuberculosis it is possible to detect the tubercle bacilli in the deposit. The fallacies are twofold. In the first place, no acid-fast bacilli may be detected at all, and yet there may be a tuberculous lesion; the proportion of error due to this cause may be lessened by having several examinations of the urine carried out upon successive days. It may incidentally be remarked that the absence of pus from urine is no absolute proof that no tubercle bacilli are present; the latter have sometimes been detected with certainty in cases where there were no pus cells present at the same time. In the second place, tubercle bacilli are not the only acid-fast organisms that the urine may contain; smegma bacilli are well-known examples to the contrary. It is true that an experienced microscopist can nearly always distinguish between these two; nevertheless, this is a very real source of error.

It becomes necessary, therefore, in a certain number of otherwise doubtful cases to inject a quantity of suspected urine into a healthy guinea-pig; this will cause the animal to die of general tuberculosis within a few weeks, even if the number of organisms is too small to be detected by direct staining. One of the chief objections to this method is the time which must elapse between the injection and the diagnosis. It has the great advantage, however, of determining the presence of tubercle bacilli even in very early cases of tuberculosis of the kidney. Like all laboratory methods, of course, it is open to errors in technique, but in a good bacteriologist's hands it is very sure. All that is required is a catheter specimen of urine collected aseptically in an absolutely aseptic bottle. The latter is conveyed, asceptically sealed, to the bacteriological laboratory, where the rest of the procedure is performed by the bacteriologist.

